# A Case of Concurrent Riedel's, Hashimoto's and Acute Suppurative Thyroiditis

**DOI:** 10.1155/2009/535974

**Published:** 2009-08-11

**Authors:** I. Pirola, M. L. Morassi, M. Braga, E. De Martino, E. Gandossi, C. Cappelli

**Affiliations:** ^1^Department of Medical and Surgical Sciences, Internal Medicine and Endocrinology Unit, University of Brescia, 25100 Brescia, Italy; ^2^Department of Pathology, University of Brescia, 25124 Brescia, Italy; ^3^II Division of General Surgery, Spedali Civili, 25124 Brescia, Italy

## Abstract

Riedel's thyroiditis (RT) is a rare form of infiltrative and inflammatory disease of the thyroid, first described by Bernard Riedel in 1896. The concurrent presence of RT and other thyroid diseases has been reported, but, the association of RT with Hashimoto's thyroiditis and acute thyroiditis has not yet been reported. We present a case of concurrent Riedel's, Hashimoto's and acute thyroiditis that occurred in a 45-year-old patient.

## 1. Introduction

Riedel's thyroiditis (RT) is a rare form of infiltrative and inflammatory disease of the thyroid, first described by Bernard Riedel in 1896 [1]. Although initially classified as a condition of primary thyroid inflammation, RT is the clinical polymorphic manifestation of a systemic multifocal fibrosclerosis disease that may involve others organs [2, 3]. 

The concurrent presence of RT and other thyroid diseases has been reported [3–6], but, to our knowledge, the association of RT with Hashimoto's thyroiditis and acute thyroiditis has not yet been reported. 

We describe a case of concurrent RT, Hashimoto's thyroiditis, and acute thyroiditis occurring in a 45-year-old patient. 

## 2. Case Report

A 45-year-old male was referred to our department for an unexplained onset of cervical discomfort and painful swelling in the left anterior area of the neck. The patients complained of acute febrile episode (39–40°C) two weeks before our evaluation, treated by ceftriaxone (2 grams daily). Physical examination showed the presence of goitre, predominantly in the left lobe, characterised by the presence of very firm tissue. The laboratory findings are reported in Table 1.

Neck ultrasound showed an enlarged thyroid left lobe characterised by the presence of a dishomogeneous marked hypoechoic tissue surrounding a hyperechoic nodular lesion with antero-posterior diameter of 15 mm and transverse of 16 mm. Both areas were promptly submitted to fine needle aspiration cytology (FNAC). During FNAC, the hypoechoic tissue felt remarkably harder than the hyperechoic nodule. 

The cytology of the hypoechoic tissue showed blood and histiocytes whereas that of the nodule showed necrotic material and polymorphonuclear leukocytes. 

Taking in account the cytological result, and moreover, the increasing symptoms of mechanical complaints such as dysphagia and dyspnea reported to the patient, he was submitted to total thyroidectomy. 

The surgeon found an extremely firm thyroid tissue adhered to adjacent structures including parathyroid glands, trachea, neck vessels, and perithyroid muscles, making dissection very difficult. 

**Figure 1:**
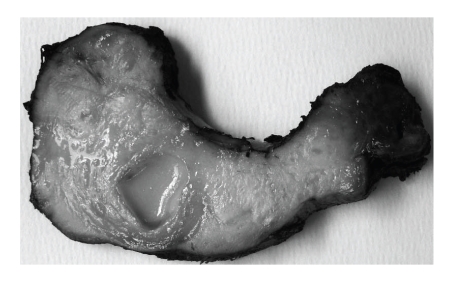
Enlarged left lobe of the thyroid with a cyst containing infected purulent material.

On gross examination, the gland was found to be asymmetrically enlarged with adherent fibroadipose and muscular tissue on the left. The cut surface was pale grey to white, firm, and fibrotic, with a cystic area containing purulent exudate (Figure 1).

**Figure 2:**
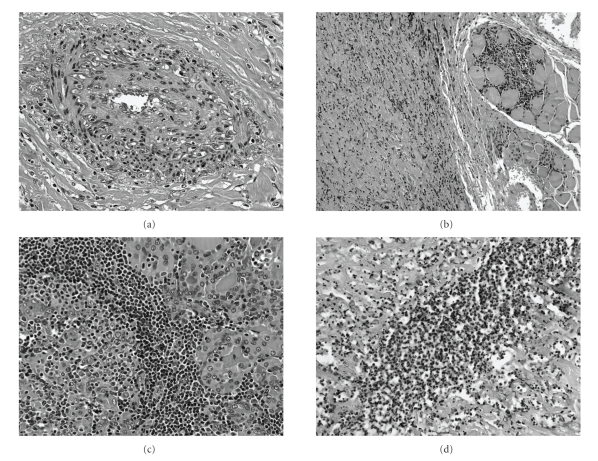
(a) “Subocclusive vasculitis” with inflammatory infiltrate in the thickened vessel wall. Note the keloid-like fibrosis around the vessel (hematoxylin and eosin, original magnification ×20). (b) Fibrosis extending into the perithyroidal skeletal muscle (hematoxylin and eosin, original magnification ×10). (c) Small follicles with scant colloid and small islands of oncocytic epithelium surrounded by an intense lymphoid infiltrate with germinal centers (hematoxylin and eosin, original magnification ×40). (d) Microabscess in a dense fibrous background (hematoxylin and eosin, original magnification ×40).

Histopathologic examination showed that the left lobe, the isthmus, and roughly the right lobe were destroyed and extensively replaced by a dense, inflamed fibrous tissue, which involved blood vessels, and extended into the perithyroidal soft tissue (Figures 2(a)  and 2(b)), encasing one lymph node and one parathyroid gland. 

In the right lobe, thyroid follicles were almost completely replaced by a dense nodular chronic inflammatory infiltrate with germinal centers. Residual follicular epithelium was atrophic with extensive oncocytic metaplasia (Figure 2(c)). Finally, the cystic area was an abscess with cellular debris, neutrophils, occasional eosinophils and histiocytes. 

Microabscesses were seldom seen in the close parenchyma (Figure 2(d)).

Immunostains for CD20, CD3, and immunoglobulin light chain showed a mixed and polyclonal pattern of B-cell and T-cell phenotype which excluded extranodal marginal zone B-cell lymphoma. The absence in the dense fibrous stroma of cytokeratin stains for AE1, AE3, CAM5.2, Ck7, panCk (Ck5,6,8, and 17), and Ck19 excluded paucicellular variant of anaplastic thyroid carcinoma or a diffuse sclerosing variant of papillary carcinoma. A solitary fibrous tumour was excluded by the absence of reactivity for CD34 and bcl-2. 

Our diagnosis was *combined Riedel's disease and fibrosing Hashimoto's thyroiditis showing coexisting suppurative thyroiditis*.

He was submitted to a whole body tomography with contrast enhancement that excluded a multifocal fibrosis involvement. 

Now the patient is in good health, undergoing thyroid replacement therapy (levothyroxine 100 mcg/daily).

## 3. Discussion

Fibrosing variant of Hashimoto's thyroiditis is characterised by extensive fibrous proliferation without extension into the surrounding structures, thus distinguishing this lesion from Riedel's thyroiditis [2, 6]. The latter is a rare disease with a prevalence of 0.05 percent or less in surgical thyroid series, described alone or in association with other thyroid diseases including thyroid cancer [3–5]. RT is characterised by the progressive fibrous replacement of the thyroid gland, with a spread of dense, inflamed fibrous tissue outside the thyroid capsule, involving the adipose tissue, muscle, and nerves that may encase vessels, parathyroid glands, trachea, and oesophagus [2, 4]. Evidence of vasculitis (predominantly a phlebitis) may be present [2, 5]. The gland is typically stone hard, and this feature may lead to clinical suspicion of carcinoma. Its aetiology remains controversial. It has been hypothesised that RT may result from an autoimmune process or from a primary fibrotic disorder owing to its association with multifocal fibrosclerosis. This uncommon idiopathic syndrome is characterised by fibrosis involving multiple organ systems such as retroperitoneal fibrosis, mediastinal fibrosis, orbital pseudotumour, pulmonary fibrosis, sclerosing cholangitis, lacrimal gland fibrosis, and fibrous parotitis: Riedel's thyroiditis may be one manifestation of this multifocal disease [2, 7]. 

On the other hand, it has been shown that detectable levels of thyroid antibodies (anti-TPO and anti-Tg antibodies) are present in some patients affected by RT, suggesting a link between RT and Hashimoto's thyroditis [8]; going further, RT might represent a rare outcome of lymphocytic autoimmune process [8]. However, currently it is generally believed that these are two different and separate entities and should be distinguished from each other on the basis of their unique clinical-pathologic features [2].

Recently the case of a patient affected by RT and Epstein Barr virus (EBV) has come to light, marking the infection as another potential trigger for the development of the thyroid disease. Indeed, the authors hypothesised that the EBV infection could be responsible for the development of recurrent process of inflammatory subacute thyroiditis, followed by the development of RT [4]. 

The association of this rare entity with other infections, such as acute suppurative thyroiditis (AST), has not been reported. AST is an uncommon thyroid disease arising from the infection of the gland by different pathogens, frequently Staphylococcus aureus, mimicking a laryngeal or pharyngeal injury, which may become life-threatening [9]. 

AST has been correlated with the presence of pre-existing piriform sinus fistula or thyroglossal duct remnant, following injury of the neck and fine needle aspiration cytology or due to septic emboli derived from infective endocarditis [10]. 

The pathological features of the present case showed the involvement of the thyroid by RT and HT and acute suppurative thyroiditis. The pathologic differential diagnosis included undifferentiated thyroid carcinoma, lymphoma, and solitary fibrous tumour. The lack of atypia and the negative cytokeratin immunostains in the spindle cells proliferation ruled out the possibility of paucicellular variant of anaplastic carcinoma, while the cellular and architectural features and immunophenotype analysis ruled out a lymphoma. Finally, the solitary fibrous tumour is circumscribed and lacks the inflammatory infiltrate of thyroiditis, and immunohistochemical studies for CD34 and bcl-2 are positive.

We report the first case of a patient simultaneously affected by Riedel's, Hashimoto, and suppurative thyroiditis. 

## Figures and Tables

**Table 1:** Laboratory findings.

Laboratory findings	Value (normal range)
C-reactive protein (CRP)	25 (<5 ng/mL)
Erythrocyte sedimentation rate (ESR)	61 (2–37 mm/h)
White blood cell count (WBC)	12.7 (4.0–10.8 10^3^/*μ*L)
Neutrophils	80.8 (40.0–74.0%)
Hemoglobin (Hb)	16.4 (14.0–18.0 g/dL)
Platelet count (PLT)	410 (130–400 10^3^/*μ*L)
Fibrinogen	510 (170–410 ng/dL)
Thyrotropin (TSH)	3.76 (0.10–5.20 mU/mL)
Free-Thyroxine (Ft4)	15.30 (7.00–20.00 pg/mL)
Anti-thyroperoxidase	>1000
Calcitonin	1.3 (0.4–18.9 pg/mL)
*β*2-Microglobulin	1.93 (1.70–2.30 mg/L)
